# On the Segmental Dynamics and the Glass Transition Behavior of Poly(2-vinylpyridine) in One- and Two-Dimensional Nanometric Confinement

**DOI:** 10.1021/acs.jpcb.1c01245

**Published:** 2021-05-28

**Authors:** Roksana Winkler, Aparna Beena Unni, Wenkang Tu, Katarzyna Chat, Karolina Adrjanowicz

**Affiliations:** † Institute of Physics, University of Silesia, 75 Pulku Piechoty 1, 41-500 Chorzow, Poland; ‡ Silesian Center for Education and Interdisciplinary Research (SMCEBI), 75 Pulku Piechoty 1a, 41-500 Chorzow, Poland

## Abstract

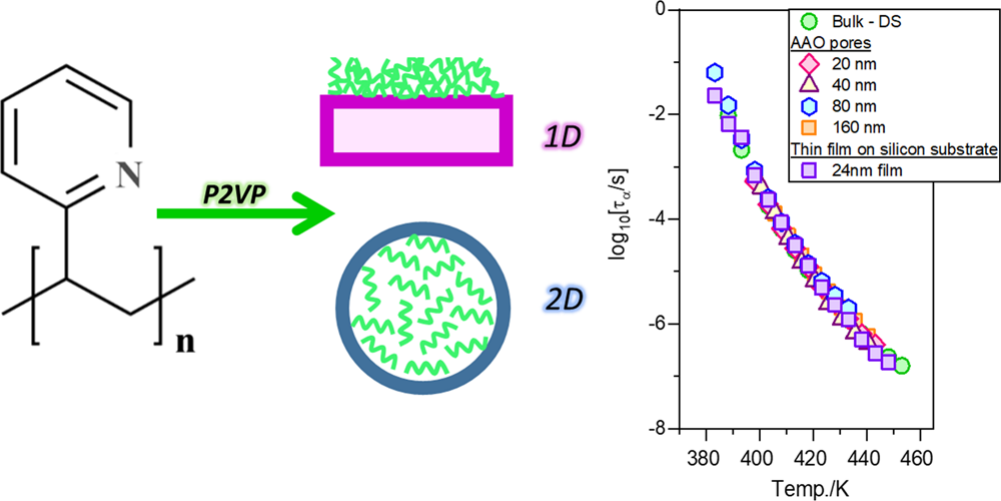

Geometric nanoconfinement, in one and two dimensions, has a fundamental influence on the segmental dynamics of polymer glass-formers and can be markedly different from that observed in the bulk state. In this work, with the use of dielectric spectroscopy, we have investigated the glass transition behavior of poly(2-vinylpyridine) (P2VP) confined within alumina nanopores and prepared as a thin film supported on a silicon substrate. P2VP is known to exhibit strong, attractive interactions with confining surfaces due to the ability to form hydrogen bonds. Obtained results show no changes in the temperature evolution of the α-relaxation time in nanopores down to 20 nm size and 24 nm thin film. There is also no evidence of an out-of-equilibrium behavior observed for other glass-forming systems confined at the nanoscale. Nevertheless, in both cases, the confinement effect is seen as a substantial broadening of the α-relaxation time distribution. We discussed the results in terms of the importance of the interfacial energy between the polymer and various substrates, the sensitivity of the glass-transition temperature to density fluctuations, and the density scaling concept.

## Introduction

Over the last few decades, there has been a growing interest in studying the properties of glass-forming materials subjected to geometrical confinement in the nanometer range. These investigations aim to understand better the basic rules governing the glass transition when the system size is comparable to the characteristic length scale associated with such a phenomenon. Recognizing the glass transition dynamics at the nanoscale level also has significant relevance for many technological applications (e.g., photoresistors, smart coatings, adhesives, biosensors, drug delivery systems, flexible organic displays, bendable electronics, and so on).^1−4^ The key features of novel nanomaterials or functional nanodevices rely on the unique physicochemical properties of the molecular systems confined within such small nanometric size dimensions that, in many cases, are substantially different from the intrinsic bulk behavior.^5−8^

Among the various available configurations, we can distinguish soft or hard confinement imposed in one (thin films), two (nanopores), or three (nanospheres) dimensions.^9−11^ Numerous studies have shown that the molecular mobility associated with the glass transition undergoes dramatic changes when reducing the size to the nanometer range.^12−14^ Changes in the glass transition dynamics include an increase, a decrease, or no effect on the characteristic time scale associated with α-relaxation.^10,15−19^ The other characteristic feature of geometrically confined glass-forming systems is a large gradient in dynamics.^20−22^ This includes retarded mobility near the confining surface/substrate and faster dynamics once moving away from the boundary interface. In nanoscale confinement, the surface energy,^23^ melting/freezing temperature, and overall phase transition behavior might strongly deviate from the bulk.^17,24−27^ Except for the finite size effect—associated with reducing the space available for the molecular arrangement—interfacial interactions between the confined molecules and the pore walls/solid substrate have a critical influence on the change of the physical properties of spatially constrained materials.^5,28−32^

Although frequently addressed in the literature, glassy dynamics at the nanometer length scale are far from being completely understood. One of the critical problems is recognizing why some glass-forming systems, including low-molecular-weight liquids and long-chain polymers, are extremely sensitive to confinement effects. In contrast, the others show bulk-like characteristics down to a few nanometers in film thickness or pore size. *Can we predict it or rationalize it in some way?* Some results indicate that molecular liquids and polymers confined in nanopores vitrify under constant volume conditions instead of constant pressure.^33−35^ For example, Zhang and co-workers demonstrated that lowering the glass transition temperature of the molecular systems embedded within the nanopores is associated with the buildup of the negative pressure.^36^ On the other hand, some other reports indicate that the pressure alone has no significant effect on the change of the glass transition temperature in nanoconfinement.^37^

Various approaches and correlations were investigated to predict or identify some basic parameters controlling changes in soft matter dynamics under nanometer confinement. For example, studying a series of polymers confined within self-ordered alumina nanopores, Alexandris *et al*. have demonstrated a trend for decreasing the glass transition temperature relative to the bulk with increasing polymer/substrate interactions.^38^ Talik *et al*. have found that enhanced dynamics in nanopore confinement correlate with the wettability, surface tension, and interfacial energy induced by increasing the surface curvature in pores of the lowest diameters.^39^ The surface energy can affect the *T*_g_ values in different ways; in case of the materials confined in the nanoporous templates, there is a trend for a decreasing glass temperature relative to the bulk with increasing interfacial energy.^38,40^ On the other hand, as reported for polymer thin films, the increase of interfacial energy can lead to an increase in *T*_g_ values so that they become higher than the bulk values.^32^

Apart from that, it has been demonstrated that perturbation in the density introduced by geometrical nanoconfinement is responsible for enhanced dynamics in nanopores^41,42^ or thin polymer films.^43^ Therefore, one can use information from the high-pressure studies of bulk materials to predict dynamics under confinement.^40,44−46^

The other problem, related mainly to thin-film dynamics, is that depending on the preparation/processing conditions, even for the same material, opposite effects can be observed in the confined state.^15,47−49^ This implies that various factors affect out-of-equilibrium glassy dynamics at the nanometer length scale.

As highlighted in a series of recent investigations on confined polymers, there is a decoupling between the calorimetric glass transition temperature *T*_g_ and the α-relaxation dynamics. In such cases, when lowering the sample size, a clear reduction of *T*_g_ is observed, whereas the molecular mobility still exhibits the bulk-like behavior.^50−53^ Some of the explanations of this finding consider the presence of interfaces, geometrical factors, and the residual stress present in the confined polymer systems.

In the context of the abovementioned aspects related to the glassy dynamics in the confined space, herein, we have examined the behavior of the polymer system poly(2-vinylpyridine) (P2VP) embedded within cylindrical alumina nanopores and prepared as thin films on silicon wafers. Inorganic nanoporous membranes and flat substrates with nondeformable solid walls as the boundary conditions provide ″hard″ nanoconfinement geometries in two and one dimensions for the polymer systems, respectively. P2VP was chosen for this study because it exhibits very favorable attractive interactions with hydroxyl groups naturally occurring on silica and alumina surfaces. Based on the literature results, the effect of nanoscale confinement on the dynamic glass transition of P2VP remains not very clear. For example, Serghei *et al*. demonstrated that the thin-film segmental dynamics of P2VP with the free upper interface prepared on ultraflat silicon wafers (rms <0.5 nm) show bulk-like behavior down to a film thickness of ∼10 nm.^54^ Likewise, no significant changes in the molecular dynamics of P2VP were found upon capillary flow through 18 nm in size cylindrical nanochannels.^55^ The segmental mobility of (semi-)isolated P2VP chains was also found to be bulk-like. Nevertheless, the broadening of the α-relaxation peak was observed.^56^ Ultrasensitive differential scanning calorimetry also shows no dependence of the glass transition temperature for P2VP films over the thickness range from hundreds of nanometers down to 3 nm.^57^ In contrast to that, Madkour *et al*. demonstrated that while the dynamic glass transition temperature for P2VP remains bulk-like for ∼22 nm films, a more detailed analysis of the calorimetric results gives an argument for a decrease in *T*_g_ with decreasing thickness—even by 7 K— as due to the presence of a mobile surface layer.^58^ Assuming strong interactions of the polymer segments with the substrates’ surface (either silica or aluminum), an increase of *T*_g_ for P2VP was also reported.^59−62^ On the other hand, Glor *et al.* showed that decoupling between molecular mobility at the free surface and near the substrate produces two distinct *T*_g_’s in ultrathin films of P2VP.^63^

Since each segment of P2VP carries a dipole moment (effective dipole moment ∼1.2 D),^64,65^ we have utilized dielectric spectroscopy to probe its relaxation dynamics associated with the glass transition. The dielectric relaxation study in a 2D-confined space was complemented by standard and temperature-modulated differential scanning calorimetry (DSC) measurements, which provide complementary information on the relaxation processes from the analysis of the frequency-dependent heat capacity response. Our results show no evidence of the confinement effect in the temperature evolution of the segmental relaxation times. This feature was observed for P2VP confined in nanoporous alumina templates with the pore size of 20 nm and 24 nm thin films supported on silicon substrates. In contrast to the bulk-like behavior of τ_α_(*T*), we observed a pronounced broadening of the segmental relaxation peak in confined geometry. We also found no evidence of decoupling between molecular mobility and the glass transition temperature. Interestingly, the broadening of the α-loss peak is not the same for 1D- and 2D-confined P2VP. Namely, the α-relaxation peak at the low-frequency side is slightly broader in the case of the nanopore-confined sample. By characterizing the strength of the interaction between the polymer and the confined surface—alumina or either silicon—we found that they are both favorable. However, P2VP is expected to have more than two times higher interfacial energy with AAO than with silicon oxide surface. To rationalize the bulk-like characteristics of the mean α-relaxation time for P2VP constrained at the nanoscale, we have taken advantage of the information that comes from the high-pressure studies of the bulk material, especially the importance of temperature and density fluctuations on the segmental relaxation of various polymer systems.^66^ This reasoning also comes from the recent experimental finding demonstrating the connection between 1D and 2D constrained polymer dynamics and bulk behavior via the density scaling approach.^44^

## Experimental Section

### Materials

**Figure 1:**
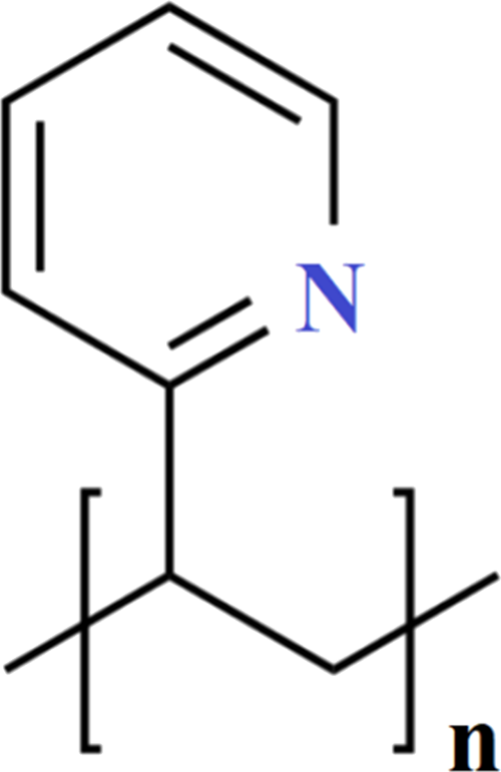
Chemical structure of the poly(2-vinylpyridine) (P2VP).

The tested sample is poly(2-vinylpyridine) labeled in the text as P2VP with the averaged molecular weight of *M*_w_ ∼54k and PDI 1.43, determined by gel permeation chromatography (GPC). The chemical structure of P2VP is given in Figure 1. The sample was purchased from Polysciences Inc. (US) as a white powder and used without further purification. The glass transition temperature of the bulk polymer determined from the DSC measurements is 375 K (recorded on a second heating run, after cooling from 423 to 293 K with 10 K/min). The literature value for P2VP with *M*_w_ of 1020k and PDI 1.33 is 373 K.^58^ The common convention to determine the glass transition temperature from the dielectric studies is to extrapolate τ_α_(*T*) to 100 s. By doing so, we get 366 K, which agrees with the value 366.8 K reported by Papadopoulos *et al.* for P2VP with *M*_w_ of 30k and PDI 1.04.^65^ However, in this study, we use the value of *T*_g_ = 372 K (defined as a temperature at which τ_α_ = 10 s) to avoid large extrapolation and for consistency with the high-pressure data.

## Preparation of Samples

### 2D Confinement

#### Nanoporous Alumina Templates and Infiltration Method

As confining templates, we have used commercially available anodized aluminum oxide (AAO) membranes (Inredox, US) composed of uniform arrays of unidirectional and non-cross-linking nanopores (pore diameter of 20, 40, 80, and 160 nm; pore depth 100 μm). The diameter of the alumina membrane is 13 mm, and its thickness is 100 μm. The porosity of AAO templates used in this study varies from 16% (for 160 nm pores) to 12% (for 20 nm pores). Before filling, AAO membranes were dried at 473 K in a vacuum oven for 24 h to remove any volatile impurities from the nanochannels. Membranes were weighed before and after infiltration. Because of the very high value of the glass transition temperature and chances to decompose the sample upon melting, P2VP was infiltrated into AAO nanopores via the solvent-assisted vapor swelling method, which has been successfully tested for numerous ultraviscous polymeric materials with reduced thermal stability.^67,68^ For that purpose, a polymer powder was placed on top of the AAO membrane and then moved to a desiccator containing a few milliliters of dichloromethane on the bottom. In the presence of dichloromethane’s vapor, P2VP softens and infiltrates into nanochannels by capillary forces even at room temperature. To achieve high filling rates, the process was carried out for a period of 2 weeks. Every few days, the membranes were taken out of the sealed desiccator and weighed. Each time, their top and bottom surfaces were carefully cleaned from the excess material using delicate wipes soaked in dichloromethane. The infiltration process was repeated until the mass of the AAO membrane with a confined polymer inside did not change with time. Finally, their surfaces were cleaned again. The membranes were put in a vacuum oven overnight (*T* = 473 K) to remove the residual solvent and weighed thereafter. The estimated degree of filling was calculated by considering the porosity of the membrane, the density of the bulk polymer, and the mass of the template before and after infiltration. The degree of pore filling varies from, in 160 nm pores, ∼60% up to ∼90%, for a pore diameter of 20 nm and 80 nm.

### 1D Confinement

#### Thin-Film Preparation

For the supported thin films, a heavily doped 4″ diameter silicon wafer (SIL’TRONIX, France) with a resistivity value in the range within 0.001–0.003 Ω·cm and orientation of (100 ± 0.5°) was used as the substrate. The wafers were diced into pieces of dimension 1 × 1 cm^2^ and were cleaned using air plasma treatment for 20 min. We used the Henniker Plasma HPT-100 with a power of 98% with 10 sccm ambient airflow for the same. Thin films were then prepared by spin-coating the polymer solution onto the cleaned silicon wafer substrate. The polymer solution, with a mass concentration of 4 g/L, was prepared in anhydrous toluene (99.8%), supplied by Sigma-Aldrich. After, it was filtered, by a 0.2 μm PTFE syringe filter, and used in a film thickness ∼24 nm. For better polymer dissolution in the solvent, we waited for 24 h before the spin coating of the polymer films onto the Si substrate. We used the KLM SCC-200 spin coater with a rotation speed of 2000 rpm, and the time was kept at 60 s. Prepared films were then annealed at 389 K for 24 h under vacuum. This procedure leads to the formation of homogeneous thin films. The film thickness was measured with spectroscopic ellipsometry and confirmed by atomic force microscopy (AFM).

#### Ellipsometry

The spectroscopic ellipsometer Semilab SE-2000 was used to measure the thin-film thickness. The measurements were done at incident angles of 65, 70, and 75° at ambient conditions. A multilayer model consisting of the Si substrate, SiOx layer, and polymer film was considered. The SiOx layer thickness before the spin coating polymer was measured and is fixed while considering the multilayer model. The thickness was obtained by fitting the ellipsometric angles and bulk material optical constants.

#### Atomic Force Microscopy

The film thickness measurement was reconciled with JPK’s NanoWizard 3 NanoScience AFM in the air using a tapping mode and a silicon cantilever. The thickness was estimated by making a scratch using a soft pen on the polymer film and measuring the step’s height. The image analysis was done using the WSxM and Gwyddion software.

## Methods

### Differential Scanning Calorimetry

Calorimetric measurements of nanopore-confined P2VP were carried out using a Mettler-Toledo DSC apparatus equipped with a liquid nitrogen cooling accessory and an HSS8 ceramic sensor (heat flux sensor with 120 thermocouples). Temperature and enthalpy calibrations were performed by using indium and zinc standards. Crucibles with prepared samples (crushed membranes containing confined P2VP) were sealed and cooled down to 293 K at the rate of 10 K/min. DSC thermograms were recorded on heating at the rate 10 K/min over a temperature range from 293 to 473 K. *T*_g_ values were determined as the point corresponding to the midpoint inflection of the extrapolated onset and end of the transition curve.

### Temperature-Modulated Differential Scanning Calorimetry

For the analysis of the dynamic behavior of the sample in the frequency range from 5 to 25 MHz, we have used temperature-modulated differential scanning calorimetry (TMDSC). Measurements were carried out at a heating rate of 2 K/min and a pulse amplitude of 1.5 K within the temperature range 343–403 K.

### Dielectric Spectroscopy

Dielectric relaxation studies for bulk and nanoconfined P2VP were carried out using a Novocontrol Alpha Analyzer. For bulk P2VP, we use standard plate–plate electrodes of 20 mm in diameter separated by a Kapton spacer of 50 μm thickness. Nanoporous AAO templates (of 100 μm thickness and 13 mm diameter) filled with the investigated polymer were placed between two circular electrodes (diameter: 10 mm). Bulk and 2D-confined materials were measured as a function of temperature (on cooling from 443 to 373 K with the rate of ∼0.2 K/min) in the frequency range from 10^–1^ to 10^6^ Hz. The temperature was controlled with stability better than 0.1 K by the Quatro system.

One should remember that the raw dielectric data collected for the nanopore-confined system represent a combined response of the sample, alumina matrix, and air gaps formed due to incomplete filling of the nanochannels. Therefore, correction of the raw dielectric data is required to access the definite signal of the confined polymer. This can be done according to the procedure described elsewhere.^69^ In previous works, we have demonstrated that the alumina template oneself and incomplete filling of the nanopores (filling degree ∼90%) do not affect the position of the α-peak and spectral shape for embedded glass-forming liquids and polymers (it only shifts ε″ toward higher values depending on the porosity).^70,71^ However, in our case, the degree of filling of the nanochannels with the tested polymer was—at least for those AAO membranes with larger pore sizes—much beyond that. Therefore, the dielectric data were corrected accordingly, and it was observed that there are no changes in the position of the α-relaxation peak compared with the bulk. Moreover, Alexandris *et al.*(38) reported that the air-gap correction is not relevant because it only affects the absolute value of the dielectric permittivity remaining the position of the relaxation peak maximum. Apart from that, such correction is also obligatory when evaluating the dielectric properties of nanopore-confined ionic liquids.^72,73^

For dielectric studies at elevated pressure, we have utilized a high-pressure setup, which includes the pressure chamber MVX-30 operating in the temperature range from 293 up to 523 K and pressures of 0–200 MPa (Unipress, Institute of High-Pressure Physics, Warsaw, Poland) and a manual pump (Sitec). The pressure was exerted using silicon oil and transmitted to a pressure vessel by a system of flexible capillary tubes (Nova Swiss). The real and imaginary parts of the complex permittivity were measured within the same frequency range as the atmospheric pressure data using an impedance Alpha-A Analyzer (Novocontrol GmbH, Montabaur, Germany). The temperature was controlled by a highly dynamic temperature control system (Huber Tango). The sample was maintained between a 20 mm in diameter plate–plate capacitor with a Kapton spacer and separated from the pressure-transmitting silicon oil by tightly wrapping it with a Teflon tape.

For the dielectric measurements of thin film, the highly conductive silicon substrate on which the polymer film was spin-coated acts as the lower electrode. The 1 × 1 mm counter electrode possesses highly insulating square SiO_2_ spacers with a side length of 5 μm and height of 60 nm. Such quadratic spacers were supplied by Novocontrol Technologies GmbH (Germany). They are produced by thermal oxidation and optical lithography on the surface of conductive silicon wafers. The two wafer pieces (i.e., the one onto which polymer film was spin-coated and the second one having silica nanospacers) are brought in contact. This gives a capacitor inside which the sample material is with its upper interface free.

Similar, as in the case of nanopore confinement, corrections of the dielectric data measured using a nanostructured electrode arrangement are required to retrieve the pure dielectric response of the thin polymer film. This can be done according to the model introduced by us in a recent paper.^74^ The model allows to distinguish the confined polymer dynamics from the total dielectric signal that is affected by the contribution coming from the nonzero resistivity of the Si electrodes, the silicon oxide isolating layer, and the spacer posts/air gap between the polymer layer and the upper electrode. The results have led to the conclusion that the most severe change in the loss peak profile in such configuration is due to the air gap that in many cases is much thicker than the polymer film itself. From the analysis of the dielectric response of various polymers within the proposed model, we found that the air-gap effect correlates with the sample polarity. For an ε_∞_/ε_s_ ratio close to 1 (small Δε values), the dielectric response of the sample is not affected significantly by this specific nanostructured-electrode geometry. For the 24 nm P2VP film supported on a silicon substrate (thickness of the oxide layer: 7 nm, air gap: 60 nm, net gap dielectric constant: 1.029, and dielectric constant of the oxide layer: 3.9), the input Havriliak–Negami (HN) fitting parameters describing the shape of the relaxation process recorded at 403 K are ε_∞_=2.15, Δε = 0.49, τ_HN_ = 8.5 × 10^–4^ s, γ = 0.25, and α = 0.91. These produce a peak shift that amounts only to ∼0.15 decades. Apart from that, they do not affect the breath of the relaxation function. For larger air gaps, the limiting shift in peak position can be estimated using the expression τ_tot_ = τ_sam_ × ε_∞_/ε_s_ and should not exceed 0.5 decades (assuming that Δε≈2.5 for bulk P2VP). Nevertheless, a more pronounced broadening of the loss profile is expected for the polymer thin film in such a case.

## Results and Discussion

### Glass Transition Dynamics in Nanopore Confinement

**Figure 2:**
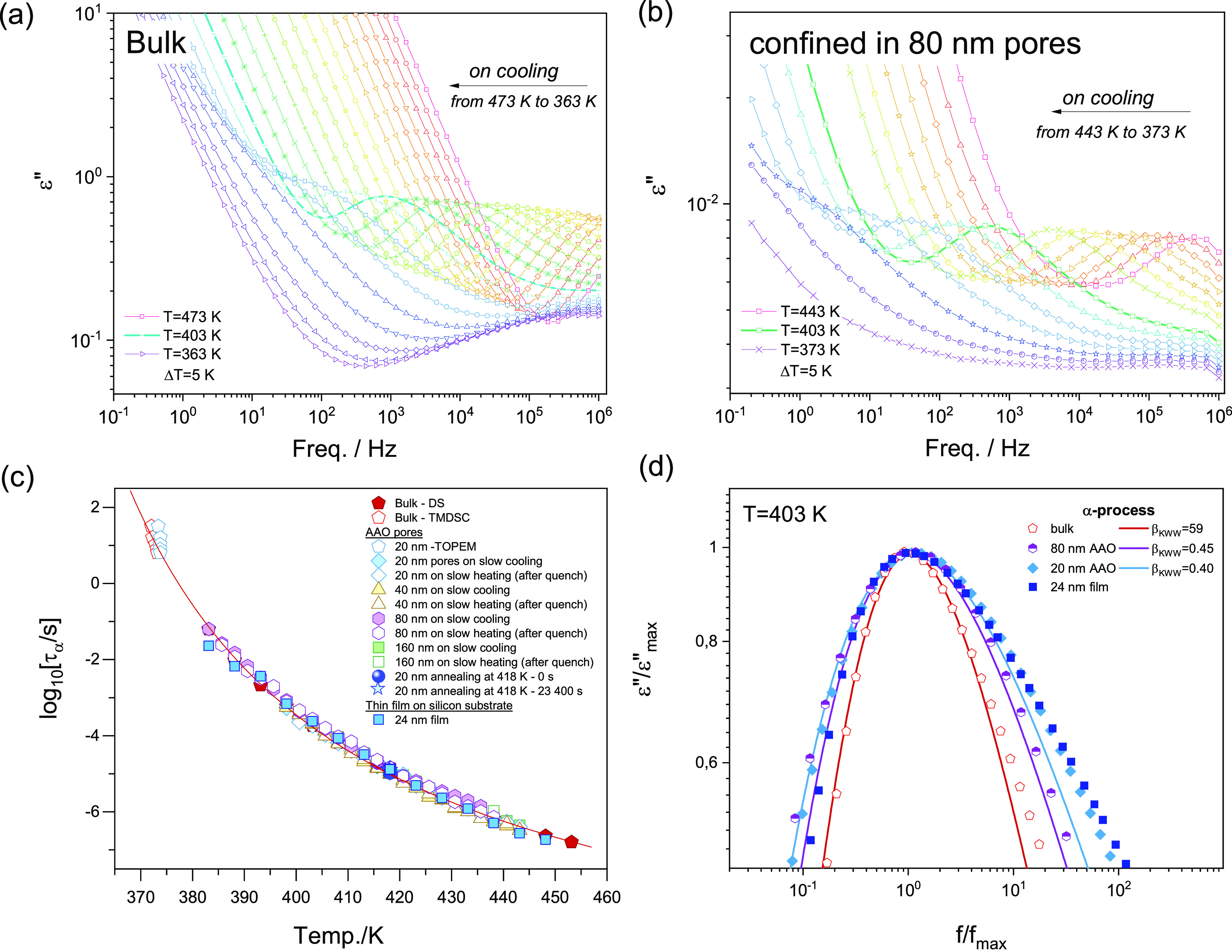
Dielectric loss spectra for P2VP (a) in bulk and (b) confined within 80 nm size alumina nanopores as measured upon cooling with the rate of 0.2 K/min. Dielectric data for nanopore confined polymer were corrected by considering nanoporous matrix permittivity and incomplete filling of the nanochannels with the polymer. (c) Temperature dependence of the segmental relaxation times for P2VP bulk (TMDSC and DS), nanopore-confined, and thin film. The red line represents the VFT fit to the dielectric data. Data in nanopore confinement were measured using two different thermal protocols: (i) on slow cooling from 443 to 383 K with the rate of 0.2 K/min and (ii) on heating with the rate of 0.2 K/min after quenching with 10 K/min from 443 to 383 K. (d) Comparison of the shape of the α-relaxation peak for P2VP confined in alumina nanopores (80 and 20 nm) and 24 nm thin film supported on a silicon substrate as measured at 403 K. Bulk spectra were given as a reference. The dc-conductivity contribution has been subtracted.

We begin our investigation by demonstrating the effect of nanopore confinement on the glassy dynamics of P2VP. The results of the dielectric relaxation studies are collected together in Figure 2. Representative dielectric loss spectra measured at the indicated range of temperatures for the studied polymer in bulk and confined within 80 nm AAO pores are shown in Figure 2a,b. In both cases, with lowering the temperature, we have observed that the main α-relaxation peak — associated with the dynamics of the polymer’s segments — shifts toward lower frequencies. This effect indicates the slowing down of the molecular movements as the glass-transition temperature *T*_g_ is approached.

The characteristic α-relaxation time is commonly determined as the frequency corresponding to the maximum of the loss peak, 1/(2π*f*_max_). However, when the dc-conductivity contribution shows up as an increase of ε″ at low frequencies, the better description of the relaxation processes is obtained by using the Havriliak–Negami (HN) function with an additional conductivity term given as:^75^
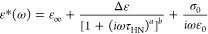
1where Δε is the dielectric strength, ε_∞_ is the high-frequency limit of the permittivity, τ_HN_ denotes the relaxation time, *a* and *b* are the shape parameters, and σ_0_ is the dc-conductivity. The characteristic time constant τ_HN_ in the HN function is related to the relaxation time at a maximum of loss peak *τ*_max_ by the following relation:^76^
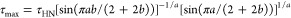
2Because of the strong conductivity contribution, not allowing to see clearly the maximum of the loss peak close to the glass-transition temperature, we have also used the derivative method (ε_der_^″^ = (−π/2)(dε″/dln*f*)≈ε_rel_^″^)^77^ that provides an alternative estimate of the α-relaxation time for bulk and nanopore-confined material. Segmental relaxation times determined for P2VP using both approaches were found to be almost the same (within the error of ±0.2–0.3 decades).

The temperature evolution of the α-relaxation times obtained in this way is shown in Figure 2c. As observed, the τ_α_(*T*) dependence for P2VP constrained within AAO templates with the pore diameter ranging from 160 to 20 nm follows bulk behavior. This contrasts to most of the glass-forming systems, which show faster dynamics with decreasing pore diameter. The segmental process for P2VP exhibits strong τ(*T*) dependence that can be described using the Vogel–Fulcher–Tammann (VFT) equation,^78,79^ as follows:
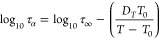
3where log_10_τ_∞_ and *D_T_* are the fitting parameters, whereas *T*_0_ is called ideal glass transition or Vogel temperature. For the investigated compound, we get the following set of parameters: −12.5, 2.4, and 315, respectively. The value of the glass transition temperature for bulk P2VP determined from the dielectric measurements is 372 K, which refers to the temperature at which the segmental relaxation times is equal to 10 s.

**Figure 3:**
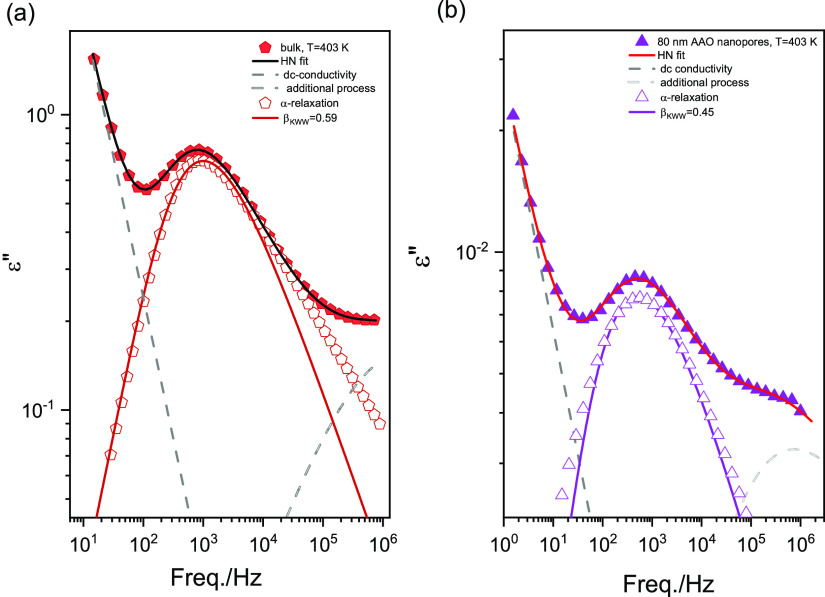
Decomposition of the dielectric loss data into the α-relaxation, conductivity contribution, and additional relaxation seen at high frequencies for bulk and nanopore-confined P2VP at 403 K.

Although we cannot actually see the effect of confinement on the temperature evolution of the mean α-relaxation time, a characteristic for nanopore-confined systems broadening of the loss peak is still observed; see the results presented in Figure 2d. This was quantified with the use of the fractional exponent β_KWW_ from the Kohlrausch–Williams–Watts function,^80,81^ as follows:
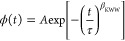
4where β_KWW_ changes from 0 to 1. The value of β_KWW_ decreases with increasing width of the relaxation spectrum. Values of β_KWW_ that describe the shape of the α-relaxation for P2VP at 403 K in the bulk state and within 20 nm AAO nanopores are 0.59 and 0.4, respectively. The broadening distribution of the relaxation times in nanopores is commonly reported and interpreted in terms of the increasing heterogeneous character of the relaxation dynamics.^19,56,82−84^ We also note that, in contrast to most glass-forming liquids and polymers confined within AAO nanopores, there is no evidence of an out-of-equilibrium behavior for nanopore-confined P2VP.^85−87^ Therefore, the shape and the characteristic relaxation time remain constant even upon prolonged annealing (see results presented in Figure 2c). Additionally, in Figure 3a,b, we demonstrate the fitting procedure used to extract the basic features of the α-relaxation for bulk and nanoconfined P2VP. The contribution from the dc-conductivity was subtracted from the total HN fit, same as the additional relaxation process seen at high frequencies (not considered in this work).

**Figure 4:**
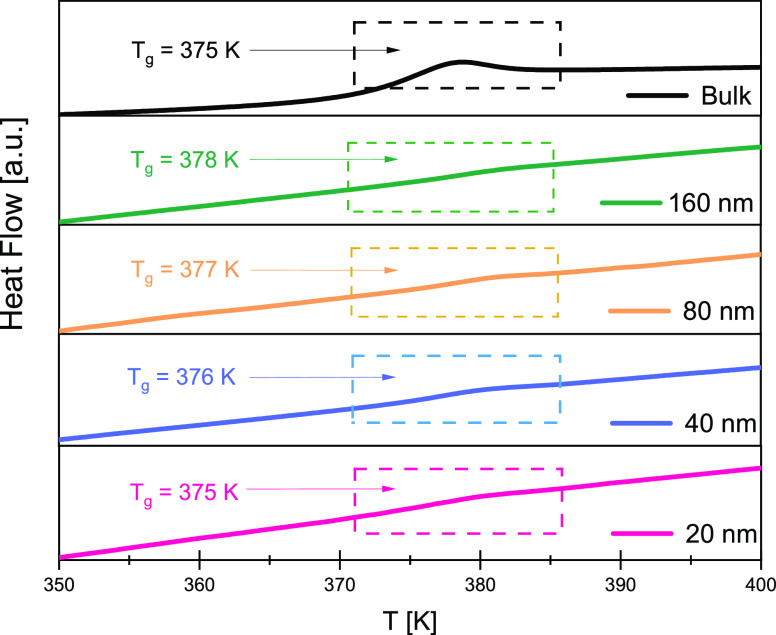
Standard DSC thermograms recorded for P2VP in bulk and confined within AAO templates of pore diameter from 160 to 20 nm. Calorimetric data were measured on heating with the rate of 10 K/min followed by quenching (10 K/min).

To validate obtained results, we have also performed calorimetric measurements that include stochastic temperature modulation (TOPEM) to determine characteristic relaxation times associated with the glass transition for bulk and P2VP confined within AAO with the pore diameter of 20 nm*.* The results again show bulk-like behavior, as demonstrated in Figure 2c. In line with this finding, upon standard DSC scans carried out for all P2VP samples constrained within 20–160 nm AAO pores, we detect only one glass transition event located close to that characteristic for the bulk polymer, i.e., at about 375 K; see Figure 4. Therefore, based on calorimetric results, we can conclude that, for P2VP, we have not observed decoupling between molecular mobility and *T*_g_ in nanopore confinement. The effect when the dynamic glass transition temperature determined from the τ_α_(*T*) does not match with calorimetric results is often observed at large confinement length scales, especially for thin polymer films.

### Glass Transition Dynamics in Thin Films

**Figure 5:**
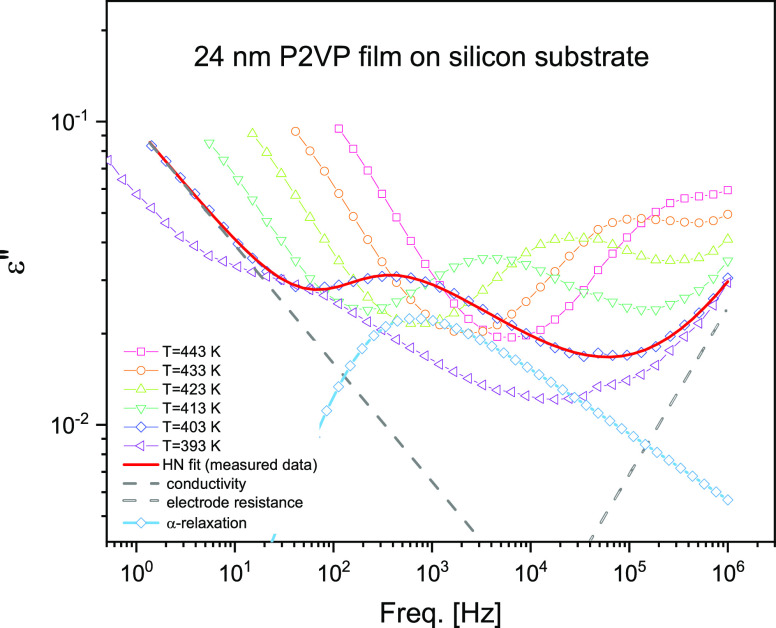
The imaginary component of the total dielectric loss response of the 24 nm P2VP thin film prepared on a silicon substrate as measured at few selected temperatures. The solid line shows the representative fit of the raw data according to the model described in our previous paper.^74^

In Figure 5, we show dielectric loss spectra collected at selected temperatures for a 24 nm P2VP film supported on silicon substrate measured using nanostructured electrode configuration. As already noted, to extract the properties of the pure polymer film, correction of the dielectric data is needed according to the model proposed by us recently.^74^ Therefore, the raw dielectric data as that presented in Figure 5 were fitted accordingly to the proposed model, assuming that the dielectric permittivity of the sample is given by the sum of the Havriliak–Negami (HN) function and a dc-conductivity term. The additional contribution seen on the high-frequency side of the spectrum is due to the nonzero resistivity of the electrodes. As we found, the value of relaxation times determined from the raw dielectric spectrum and that obtained by following the abovementioned correction deviate from each other only very slightly. This relates to the small value of the dielectric strength for the P2VP film. This is consistent with the results by Kremer and co-workers,^13,88^ who demonstrated that the shape and the mean relaxation rate of the polymer film measured in an air-gap geometry do not change for low values of dielectric strength, as opposed to the case of large strengths. Values of the relaxation times determined in this way were then added to bulk and nanopore data presented in Figure 2c. As observed from the results, the temperature dependence of the α-relaxation times for the 24 nm film follows that of the bulk polymer, whereas the breadth of the relaxation process shows only a difference from that of the 20 nm pore confined sample at the low-frequency side of the loss peak (see Figure 2d). We conclude that stretching of the polymer chains is not sufficient to alter the *T*_g_ or the cooperative segmental dynamics of P2VP in confinement. Nevertheless, the width of the α-relaxation process increases under the condition of geometrical confinement. Changes in the distribution of relaxation times reflect the increased heterogeneous character of the environment and a broadening of the glass transition.

Given the results presented above, the question arises why, for P2VP, the segmental dynamics remain bulk when going down with the pore diameter and in thin films. In contrast, for most of the glass-forming systems, we typically observe enhanced dynamics in confined geometry. Can we rationalize it as due to strong interactions (e.g., via hydrogen bonds) with the pore walls, same as expected in thin-film dynamics? On the other hand, strong interfacial interactions should retard the mobility of these polymer segments, which are located close to the pore walls. And this should actually make the gradient in dynamics between core and interfacial layers to be more pronounced. We could use the argumentation of the characteristic length scale on which the glassy dynamics occur. The results suggest that even in 20 nm pores, there are no changes in the cooperativity of the relevant molecular motions. Moreover, it is observed that even with a much higher molecular weight for other polymer systems, these changes are evident. In agreement with recent studies,^41,42,89^ changes in the molecular packing or frustration in the density are the source of the enhanced mobility of glass-forming systems in nanopores. This immediately leads to a potential role of the pressure effects^33,35,44−46^ that, together with the temperature variation, are known to be the key factors that control the glass-transition dynamics.^90^ Therefore, to better understand the bulk-like behavior of P2VP under nanoscale confinement, we have used the information that comes from the high-pressure studies of the bulk material. These results are described in the next part of this paper.

### Glass Transition Dynamics for Bulk P2VP on Increased Pressure

**Figure 6:**
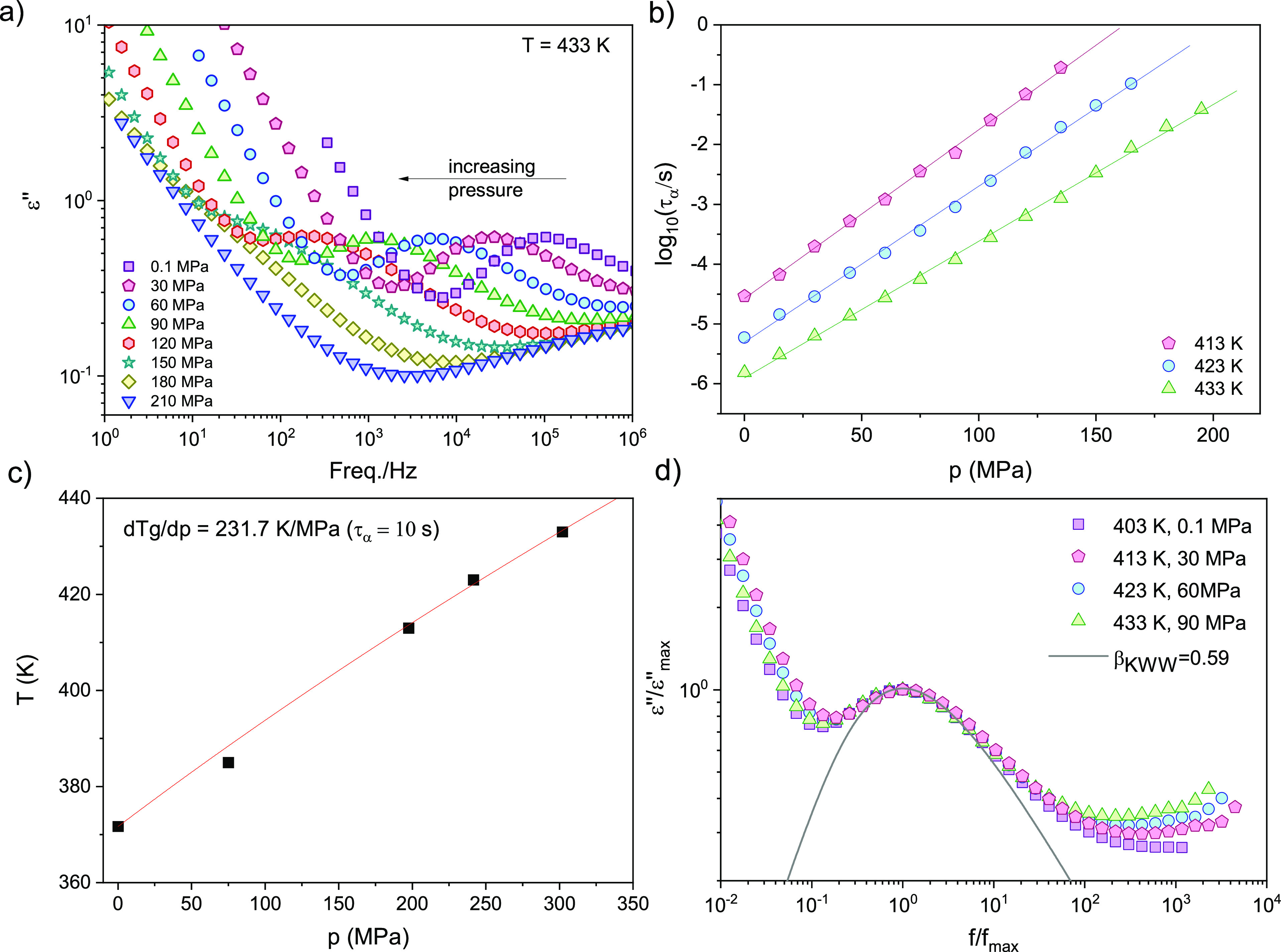
(a). Dielectric loss spectra measured for bulk P2VP along isotherm *T* = 433 K. (b) α-Relaxation time plotted versus pressure along three different isotherms. Solid lines represent fit to the data with the use of the pressure version of the Arrhenius equation. (c) Variation of the glass transition temperature as a function of pressure. The glass transition was determined from dielectric data as the temperature (pressure) at which τ_α_ = 10 s. The red line represents the Andersson–Andersson fit. (d). Comparison of the normalized dielectric loss spectra obtained at different thermodynamic conditions (*T*, *p*) for approximately the same α-relaxation time (so-called isochronal superposition plot).

Figure 6a illustrates representative dielectric loss spectra measured for P2VP at constant temperature *T* = 433 K and different pressures. With increasing pressure, the α-relaxation peak moves toward lower frequencies, indicating a systematic slowing down of the segmental dynamics. Due to the high dc-conductivity contribution, the α-relaxation time was extracted using the same procedure as in confinement data. Obtained in this way, pressure dependencies of the α-relaxation times measured along three different isotherms are shown in Figure 6b. The pressure dependencies of the segmental relaxation times have a linear character and therefore were described with the use of the pressure version of the Arrhenius law,^91^ as follows:
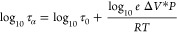
5where log_10_τ_0_ is a fitting parameter, ΔV* is the activation volume, and *R* is the gas constant. By extrapolating VFT fits to 10 s, we can determine the corresponding values of the glass-transition pressure, *p*_g_ (as the equivalent of the glass-transition temperature for isobaric data). The glass transition is usually determined from dielectric relaxation studies as the temperature/pressure at which τ_α_ reaches 100 s. Nevertheless, in this work, we have used 10 s to avoid extrapolation of the data.

To further quantify the effect of pressure on segmental dynamics of P2VP, we have calculated the pressure coefficient of the glass-transition temperature *dT*_g_/*dP* determined as the first derivative of the experimentally measured *T*_g_(*p*_g_) dependence in the limit of ambient pressure. This is presented in Figure 6c. To describe the dependence of the glass transition vs pressure, we have utilized the empirical equation proposed by Andersson and Andersson,^92^ as follows:
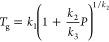
6The best fit was obtained with *k*_1_ = 371 ± 1, *k*_2_ = 2.57 ± 1, and *k*_3_ = 1603 ± 173. By dividing *k*_1_ by *k*_3_, one can estimate the value of the pressure coefficient of the glass-transition temperature *dT*_g_/*dP*, which describes the sensitivity of the glass-transition temperature to pressure changes. For P2VP, we get 232 K/GPa, a relatively low value for a polymeric system and rather more typical for a low-molecular-weight van der Waals liquids. By comparison, the values are 328 K/GPa for polystyrene (PS),^66^ 289 K/GPa for poly(methylphenylsiloxane) (PMPS),^93^ 520 K/GPa for bisphenol-A-polycarbonate,^94^ and 481 K/GPa for poly-4-chloro-styrene (P4ClS).^44^ As a matter of fact, the *dT*_g_/*dP* coefficient determined in this study for P2VP is much lower than that reported by Papadopoulos and Peristeraki,^65^ 340 K/GPa, using τ_α_ = 100 s and the sample of MW ∼31k. We suppose that the origin of such discrepancy might be the high conductivity contribution, which even made the authors choose dielectric modulus instead of dielectric permittivity representation to analyze the data obtained on increased pressure.

In agreement with recent experimental results, the *dTg*/*dP* coefficient’s value might provide a rough estimate on whether the α-relaxation process is sensitive to the density fluctuation in confined geometry. As found by Talik and co-workers,^40^ the depression of the glass transition in AAO nanopores correlates with the *dT*_g_/*dP* ratio; i.e., the higher the *dT*_g_/*dP* coefficient is, the more deviation from the bulk behavior is observed in confined geometry. Lipson and co-workers also pointed out a similar finding when trying to rationalize the enhanced dynamics of thin films of P4ClS.^45^ Likewise, the much different behavior of low-molecular-weight liquids glycerol (35 K/GPa) and salol (204 K/GPa) confined in alumina templates was explained in terms of the different contribution of volume and thermal effects in controlling their glassy dynamics.^33^ In line with this, the relatively weak sensitivity of P2VP to pressure/density effects might be the origin of its bulk-like behavior in AAO nanopores. One can also use the same type of argumentation for 1,4-*cis*-polyisoprene (*dT*_g_/*dP* = 178 K/GPa),^95^ in which glass transition temperature was unaffected by 2D confinement, whereas, at the same time, a remarkable broadening of the distribution of relaxation times for both the segmental and chain modes was observed.^96^

In Figure 6d, we plot the dielectric loss spectra measured at different combinations of (*T*, *p*) but with approximately the same α-relaxation time. To have a perfect overlap, their maxima were normalized approximately at frequency *f* = 10^3^ Hz. The best KWW fit obtained for the relaxation peak of the P2VP is β_KWW_ = 0.59. Although the dc-contribution is relatively high, it is evident that the distribution of the relaxation times for P2VP remains *T*–*p* invariant along an isochrone (isochronal superposition). Herein, we wish to note that the isochronal superposition fails for glass-forming systems that reveal strong hydrogen bonding interactions, such as dipropylene glycol.^97^

**Figure 7:**
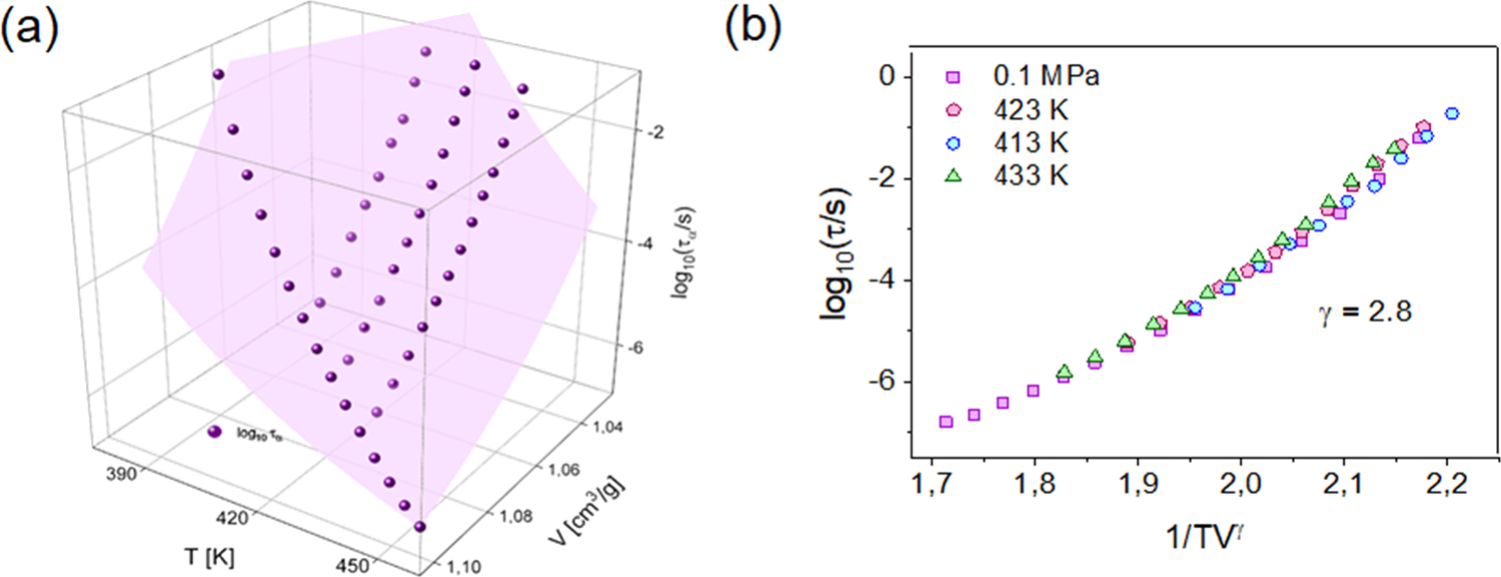
(a). α-Relaxation times plotted versus temperature (*T*) and volume (*V*) for P2VP. The violet area represents the surface fit to the modified Avramov equation. (b) Density scaling dependence of the segmental relaxation times obtained for P2VP using ambient and high-pressure dielectric data.

In the next step, with the use of PVT data reported in the literature (for P2VP of similar molecular weight),^65^ we converted experimentally measured τ_α_ (*T*, *p*) dependences to τ_α_ (*T*, *V*) ones. Then, we parameterized them with the use of the modified version of the Avramov model,^98^ as follows:
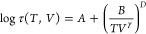
7where *A*, *B*, *D*, and γ are the fitting parameters. The two-dimensional surface plot described with the set of fitting parameters γ = 2.8, *D* = 5.5, *B* = 657, and *A* = −8.7 is presented for P2VP in Figure 7a. Then, using the value of γ = 2.8 for P2VP, we tested the density scaling, i.e., the ability to describe the relaxation time measured under varying temperature and pressure conditions as a function of the single scaling relation ρ^γ^/*T*.^99,100^ The gamma exponent found by describing high-pressure data with the use of eq 7 is a material constant, often related to many dynamic and thermodynamic properties of the system.^101−103^ Therefore, it provides key information about the intermolecular forces or dynamic behavior of the substance. For P2VP, using the scaling exponent value =2.8, we were able to superimpose the α-relaxation times measured under different isobaric and isothermal conditions presented in Figure 7b. The validity of the density scaling for P2VP indicates that its hydrogen bonding tendency is not enough to affect the glass-transition dynamics at varying thermodynamic conditions. In addition to that, the gamma exponent’s value for P2VP is typical for other polymer glass-formers (P4ClS: 3.1, PS: 2.5, and 1,4PI: 3), indicating that we cannot use it to predict any relevant information on confinement effects.

### Surface Free Energy Comparison

The presence of a hard interface is known to cause changes in the local density and affects the dynamics of the confined polymer chains. For example, Fryer *et al.* showed that the deviation in *T*_g_ from the bulk value strongly depends on the interfacial energy.^32^ The results of the molecular dynamics simulations also show that the *T*_g_ of the ultrathin polymer can be tuned by changing the intermolecular potential between the polymer chains and the substrate.^104^ Recently, Zuo et al. demonstrated that the polymer thin film dynamics can be tuned by the interfacial effects introduced by changing both the strength and degree of chain adsorption via surface modification of the substrates.^105^ Specifically, the polymer–substrate interfacial effects were affected by attaching silane reagents with phenyl and amino groups onto the surface. As reported, with decreasing surface free energy, *T*_g_ of the PMMA films also decreases and deviates more from the bulk polymer.

To understand the type of interaction between P2VP with Al/alumina and SiO_2_, we calculated the surface free energy between the polymer and the substrates. The total surface free energy of a material, γ^Total^, can be expressed as follows:
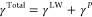
8where γ^LW^ is the dispersive and γ*^P^*is the polar component of the surface energy, respectively.^106^Table 1 shows the γ^Total^, γ^LW^, and γ*^P^*values for P2VP, Al, alumina, and SiO_2_ calculated from the contact angle values available in the literature.^58,107,108^ One can estimate the interfacial energy between two materials, in our case, the polymer ″P″ and the substrate ″S″, as follows:^109^
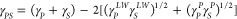
9

**Table 1:** The Interfacial Energy between P2VP and Various Substrates Calculated from the Total Surface Energy of Material (γ^Total^) along with Its Dispersive (γ^LW^) and Polar (γ*^P^*) Components

	γ^Total^ (mJ m^–2^)	γ^LW^ (mJ m^–2^)	γ*^P^* (mJ m^–2^)	γ*^PS^* (mJ m^–2^)
P2VP	39.5	29.8	9.7	
Al	28.32	26.5	1.82	3.21
SiO_2_	47	44.6	2.3	4.14
alumina	36.3	36.3	0	10

The calculated values of γ*_PS_* are also collected in Table 1. The values of γ*_PS_* show that the interaction between P2VP and solid substrates increases in the order Al, SiO2, and alumina. In all cases, P2VP possesses strong interaction toward the surface of the substrate. In agreement with the literature, for γ_SL_ lower than approximately 2 mJ/m^2^, the measured *T*_g_ should be lower from that of the bulk sample, while for γ_SL_ > 2 mJ/m^2^, an increase in *T*_g_ should be observed.^32,105^ This interfacial energy can possibly affect the broadening of the relaxation in a way that the more the interaction energy is, the more the number density of slowed down segments is at a given temperature. A keen look at Figure 2d reveals that the broadening on the low-frequency side of the α-relaxation is correlated with the interfacial energy values. This broadening (and for some polymers or geometries, a completely new separate mode) is understood to be due to an interphase formed by slowed down segments.^110−113^ Although we actually have not seen an increase of *T*_g_ for confined P2VP, the results of the above calculation, together with the high-pressure data, indicate the pronounced importance of the polymer–substrate interactions and sensitivity of the polymer dynamics on density frustrations as the potential source responsible for changes in the glass transition dynamics in nanoconfinement.

As the last point, it should be noted that free surfaces, substrate interfaces, and confinement can all together significantly perturb the glass transition dynamics of polymers confined at the nanoscale. This can lead to deviations from bulk *T*_g_ that can be either barely noticeable (as individual components will counteract each other) or difference of many degrees kelvins (when they reinforce).^20,32,114^ It is typically believed that the presence of a free surface results in the enhanced mobility of the polymer segments adjacent to the free surface. Simultaneously, attractive substrate interactions reduce the mobility of chain segments anchored to the substrate, which increases *T*_g_. Overall, this might suggest that the absence of change in *T*_g_ of P2VP in confinement comes from the conflicting effect of the attractive substrate (increase *T*_g_) and free surface (decrease *T*_g_). However, a significant problem with estimating the impact of free surface on thin-film polymer dynamics is that practically changes in *T*_g_ probed as a function of distance from the free surface or supporting substrate can be studied using fluorescent dyes or via computer simulations.^20^ As showed by Torkelson and co-workers in the case of P2VP, the free-surface effect can be negligible in dictating the *T*_g_ behavior of supported ultrathin polymer films. More specifically, it is overdominated by strong attractive interactions of the polymer hydroxyl groups with the silica substrate.^62^

## Conclusions

In this work, we have studied the segmental dynamics of P2VP in one- and two-dimension nanoconfined geometry provided by silicon substrates and porous alumina membranes. The dielectric relaxation studies, together with calorimetric results, show that nanoconfinement does not induce slowing down of the molecular motion of P2VP in AAO nanopores. The α-relaxation time displays bulk-like *T*-dependence in pores with sizes down to 20 nm. Moreover, we have also not seen deviations in τ_α_(*T*) for the 24 nm thin film supported on a silicon substrate. On the other hand, the confinement of P2VP in AAO nanopores and thin films results in a substantial broadening of the distribution of relaxation times. Interestingly, we found that the breadth of the α-relaxation time for P2VP in 20 nm size pores and 24 nm thin film does not show a substantial difference, although there is a slight broadening seen on the low-frequency side in the case of the 2D-confined sample.

To understand why the behavior of segment relaxation time for nanoconfined P2VP remains bulk-like, we have made use of the information that comes from the high-pressure studies of the bulk material. The *dT*_g_/*dP* coefficient for P2VP is 232 K/GPa, meaning that its glass-transition dynamics are rather weakly sensitive to pressure/density effects. This most probably explains why the segmental relaxation time is not affected by changes in the density induced by geometrical constraints. Not all polymers are sensitive to confinement effects, same as they are not sensitive to density fluctuations/compression. Hence, by relating these two features, we are able to envisage the potential changes in the dynamics of polymer glass-formers in nanoscale confinement. From the high-pressure studies, we also found that the value of the density scaling exponent, γ = 2.8, for P2VP is quite similar to other polymer systems of much different *dT*_g_/*dP* values. Hence, we cannot use it to explain/predict the effect of geometrical confinement on *T*_g_. The other parameter very useful to understand the properties of P2VP under nanoscale confinement turned out to be the surface free energy between the polymer and the substrates. The estimated γ*_PS_* values indicate the pronounced importance of the polymer–substrate interactions for all considered cases here. The broadening of the α-relaxation seen at low frequencies seems to correlate with the trend in the calculated surface energies. This broadening is understood to be due to an interphase formed by slowed-down segments. Therefore, by linking the strength of the interaction with the solid substrate and sensitivity of the glass-transition temperature to density variation, we can aim to rationalize the α-relaxation dynamics of the confined polymer system.
